# Single cell RNA-seq analysis reveals temporally-regulated and quiescence-regulated gene expression in Drosophila larval neuroblasts

**DOI:** 10.1186/s13064-022-00163-7

**Published:** 2022-08-24

**Authors:** Noah Dillon, Ben Cocanougher, Chhavi Sood, Michelle Xin Yuan, Andrea B Kohn, Leonid L Moroz, Sarah E Siegrist, Marta Zlatic, Chris Q. Doe

**Affiliations:** 1 Institute of Neuroscience, Howard Hughes Medical Institute, University of Oregon, OR 97403 Eugene, USA; 2 Department of Zoology, University of Cambridge, Cambridge, UK; 3 Department of Biology, University of Virginia, VA 22904 Charlottesville, USA; 4 Whitney Laboratory for Marine Biosciences, University of Florida, FL 32080 St. Augustine, USA; 5 MRC Laboratory of Molecular Biology, Dept of Zoology, University of Cambridge, Cambridge, UK; 6 Janelia Research Campus, VA Ashburn, USA

**Keywords:** Neuroblast, Intermediate neural progenitor, Temporal transcription factor, Single cell RNA-sequencing, Quiescence, Insulin signaling

## Abstract

The mechanisms that generate neural diversity during development remains largely unknown. Here, we use scRNA-seq methodology to discover new features of the *Drosophila* larval CNS across several key developmental timepoints. We identify multiple progenitor subtypes – both stem cell-like neuroblasts and intermediate progenitors – that change gene expression across larval development, and report on new candidate markers for each class of progenitors. We identify a pool of quiescent neuroblasts in newly hatched larvae and show that they are transcriptionally primed to respond to the insulin signaling pathway to exit from quiescence, including relevant pathway components in the adjacent glial signaling cell type. We identify candidate “temporal transcription factors” (TTFs) that are expressed at different times in progenitor lineages. Our work identifies many cell type specific genes that are candidates for functional roles, and generates new insight into the differentiation trajectory of larval neurons.

**Supplementary Information:**

The online version contains supplementary material available at https://doi.org/10.1186/s13064-022-00163-7.

## Introduction

A major question in neuroscience is how neural diversity is generated, which underlies complex neural circuits and behavioral output of the central nervous system (CNS). In the past, neuronal diversity was commonly defined by morphological features (axon/dendrite processes), biochemical features (neurotransmitter choice), and physiological features (distinct ion channels and membrane properties) [1]. In addition, “low throughput” assays for molecular differences among neurons, typically for transcription factor (TF) expression, have been crucial for finding insights into the generation of neural diversity for decades [2, 3]. Taken together, these approaches resulted in the definition various classes or subtypes of motor neurons, interneurons, sensory neurons and peptidergic neurons, but they are ill-suited to address the question of how many unique types of neurons exist within the CNS, and the subsequent question of how each cell type contributes to the function of the CNS.

The advent of single cell RNA sequencing (scRNA-seq) allowed a more complete inventory of gene expression profiles within individual neurons, with the expression of “validated cell type genes” used as a framework to identify transcriptionally related neurons [4–8]. Further analysis has revealed novel cell types based on common gene expression, but also that trajectories between cell types to be more gradual and less saltatory than previously appreciated, in part due to transcriptional priming [9–11].

In *Drosophila*, neuronal scRNA-seq has been done on adult brain [12–16], pupae [17–22], larvae [23–25], and blastoderm-stage embryos [26]. These experiments have provided valuable insight into the number of distinct neuronal types and identified gene candidates for regulating neural subtype function or connectivity. However, no studies to date have focused on identifying and characterizing the transcriptional diversity of neural progenitors, nor has any study mapped progenitor transcriptional profile at multiple larval stages. In this study, we identify multiple progenitor subtypes across several larval stages with differential gene expression to provide candidate genes as cell type specific markers and functional roles during development.

## Results

### Larval atlas shows distinct cell identities and differentiating neural progenitor axis

**Fig. 1:**
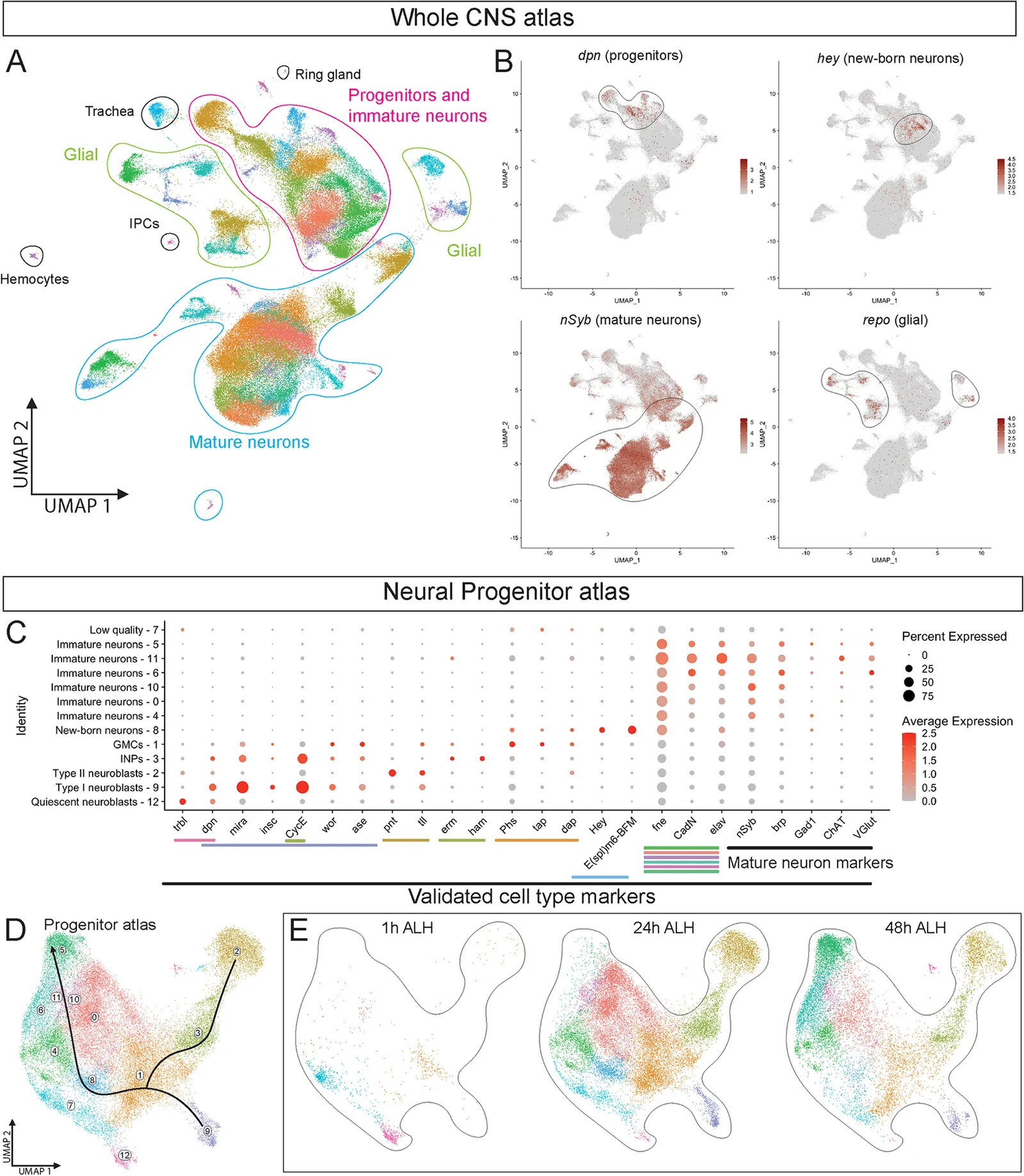
Larval atlas shows distinct cell identities and differentiating neural progenitor axis. **A** An atlas of 97,845 cells collected from 1h, 24h and 48h ALH larvae was built. These cells were analyzed with Seurat and clustered to identify major cell types such as neural progenitors, glial, mature neurons and other features to validate the atlas in clustering by cell type. **B** Feature plots of *Dpn* and *Hey* show a differentiating neural progenitor axis. The mature neuronal marker *nSyb* shows limited expression in progenitors that extends into mature neuronal clusters. The glial marker *repo* shows glial cells separated from progenitors and mature neurons. **C** Validated cell identity markers label distinct progenitor cell types within a developmental axis. **D** Progenitor atlas was made with a subset from the whole atlas of 33,458 neural progenitor and immature neuron cells. Black line indicates expected developmental trajectory. **E** UMAPs of progenitor clusters from 1h-48h ALH

To identify single cell gene expression profiles throughout larval development, we used scRNA-seq data collected by [27] from dissociated brain and ventral nerve cord (VNC) – together termed the CNS – from larvae at 1h, 24h and 48h (all times in hours after larval hatching; ALH). We used the 10X Genomics pipeline for scRNA-seq analysis and used Cell Ranger Aggregation to aggregate multiple samples from the same timepoint. We used the standard Seurat integration pipeline to filter out low quality cells and clustered 97,845 cells from all larval stages (see methods; Fig. 1a). Within our atlas we identified clusters enriched for cell types in the CNS: neural progenitors, immature and mature neurons, glia, trachea, hemocytes and insulin-producing cells (IPCs; Fig. 1a; Supp. Table 1). Representative examples of a progenitor marker (Deadpan; *dpn*), a new-born neuron marker (*Hey*), a maturing neuron marker (*nSyb*), and a glia marker (*repo*) are shown in Fig. 1b.

We next focused on the progenitor and immature neuron cluster, sub-clustering only these cells. We found clear separation of the major progenitor cell types: quiescent neuroblasts (cluster 12), type I neuroblasts (cluster 9), type II neuroblasts (cluster 2), Intermediate Neural Progenitors (INPs, cluster 3), Ganglion Mother Cells (GMCs, cluster 1), new-born neurons (cluster 8), and immature neurons (clusters 0, 4–6, 10, and 11), plus one low quality cluster (7) that was excluded from subsequent analysis (Fig. 1c; Supp. Table 2). Clusters were assigned cell type designations based on enriched expression of experimentally validated cell type markers (Fig. 1c; Table 1). Interestingly, each class of progenitor formed a distinct cluster, creating a differentiation axis right to left in UMAP space (Fig. 1d). Not surprisingly, the quiescent neuroblast cluster was enriched at 1h when most neuroblasts are quiescent [28], followed by emergence of proliferating neuroblasts, INP and GMC clusters at 24h and 48h (Fig. 1e). Thus, we can identify and transcriptionally profile all known progenitor subtypes across larval development, including quiescent neuroblasts which have never been identified in RNA-seq experiments. We discuss each progenitor type in more detail below (Tables 2 and 3).

**Table 1:** Validated markers for progenitors and young neurons

Cell type	Marker	References
Neuroblast, quiescent	Tribbles +	[29]
	Deadpan+	[29]
	Worniu -	[29]
Neuroblast, Type I	Deadpan +	[30]
	Asense +	[31]
	Worniu +	[32]
	Miranda +	[33]
	Inscuteable +	[34]
	String +	[34]
	Cyclin E +	[35]
Neuroblast, Type II	Pointed +	[36]
	Tailless +	[37]
	Asense -	[31]
INP	Erm +	[38]
	Hamlet +	[37]
	Cyclin E +	[25, 39]
GMC	Target of Poxn +	[25]
	Dacapo +	[40]
	Miranda -	[33]
Neuron, new-born	Hey +	[41]
	E(spl)m6BFM +	[25]
Neuron, immature	Elav+	[42]
	Ncad+	[43]
	Fne +	[44]
	Brp-	[45]
	nsyb-	[46]
Neuron, mature	Brp+	[45]
	nSyb+	[46]

**Table 2:** Validated markers for glial cell types

Cell type	Marker	References
All glial	Repo +	[47, 48]
Astrocyte/neuropil glial	Gat +	[49]
	Alarm +	[50]
Perineurial	CG6126 +	[51]
	Indy +	[52]
	CG4797 +	[12]
Subperineural	Moody +	[53]
	AdamTS-A +	[52]
Cortex/chiasm glial	Wrapper +	[54]
	Hoe1 +	[12]

**Table 3:** Validated markers for mature neuron cell types

Cell type	Marker	References
Undifferentiated	Hdc+	[55]
	Ncad+	[43]
Cholinergic	Ace +	[56]
	ChAT +	
GABAergic	Gad1 +	[57]
Glutamatergic	VGlut +	[58]
Monoaminergic	Vmat +	[59]
	Ddc +	[60]
	Trh +	[61]
Peptidergic	Dimm +	[62]
	CCAP +	[62]
	Burs +	[63]
	AstC +	[64]
Octopaminergic	Vmat +	[59]
	Tbh +	[65]
	Tdc2 +	[66]
Motor neurons	Twit +	[67]
Kenyon cells γ	Rgk1	[68]
	Pka (R1/2, C1)	[69]
Neurosecretory cells	ITP	[70]
	sNPF	[70]

### Quiescent neuroblasts and associated glia are enriched for expression of genes regulating the TOR and insulin pathways

**Fig. 2:**
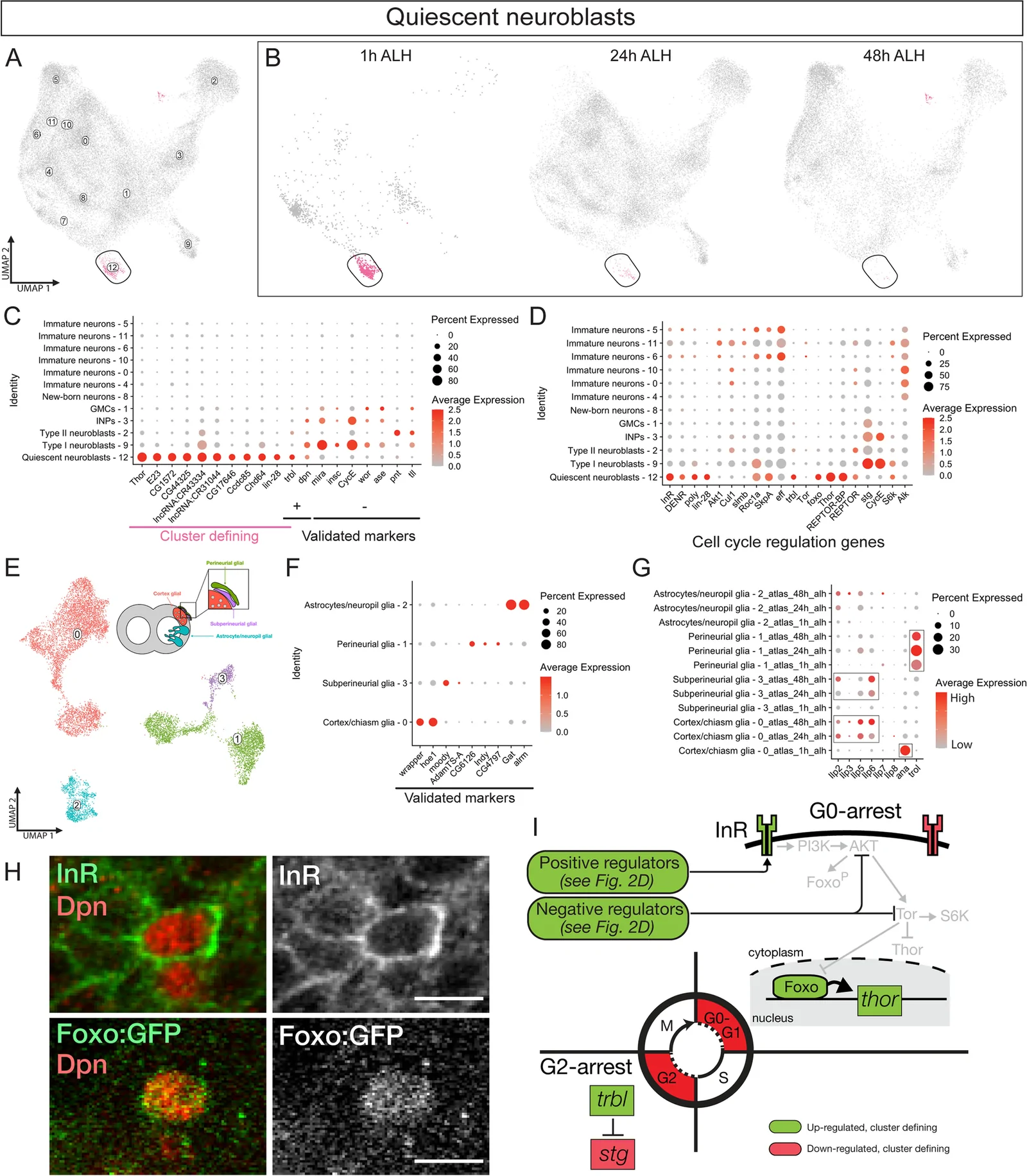
**Quiescent neuroblasts and glial cells show enriched markers for regulating the TOR and insulin pathway. A** UMAP of CNS cell types with quiescent neuroblasts in cluster 8 (circled). **B** UMAP of cluster from 1h-48h ALH. **C** Dot plot of top cluster defining genes alongside validated cell identity markers for quiescent neuroblasts. **D** Dot plot of genes involved with cell cycle regulation including the insulin signaling, AKT and TOR pathways. **E** UMAP re-clustered of 11,004 glia cells from a subset of the whole atlas. Diagram adapted from Kremer et al, 2017 [73]. **F** Validated glial cell type markers. **G** Temporal expression of signaling molecules involved in neuroblast quiescence within glial subtypes. **H** Validation of InR and Foxo expression in Dpn+ quiescent neuroblasts. Scale bar, 5 uM. **I** Model depicting cell growth and cell cycle genes identified as significantly enriched or depleted in the quiescent neuroblast cluster at 1h ALH, placed in the context of known signaling pathways

The majority of neuroblasts enter quiescence before the end of embryogenesis and remain quiescent until 12-24h [71, 72]. We noticed that cluster 12 is clearly present at 1h but the number of cells drop at 24h and 48h (Fig. 2a-b); this timing coincides with neuroblasts exiting quiescence. We confirmed this cluster 12 identity as quiescent neuroblasts using the positive markers *dpn* and *trbls* in addition to the lack of expression of canonical proliferating neuroblast markers (Fig. 2c; Table 1; Supp. Table 2). The top cluster defining genes (i.e., genes that define the cluster as a distinct grouping of cells) represented cell growth, cell cycle progression and the insulin signaling pathway (Fig. 2c).

To further investigate gene expression in this quiescent neuroblast population, we analyzed the expression of core elements regulating the insulin receptor (*InR*), AKT pathway, TOR pathway and general markers of cell growth. We were interested in InR regulation in quiescent neuroblasts because previous work has showed insulin signaling to be essential for neuroblasts to exit quiescence [28, 74]. We found upregulation of *InR* in addition to upregulation of positive regulators for *InR* (Fig. 2d). AKT and TOR genes showed lower expression (Fig. 2d), consistent with the lack of cellular growth in quiescent neuroblasts. Similarly, markers for cell cycle genes showed low expression (Fig. 2d). We conclude that quiescent neuroblasts are transcriptionally primed to receive insulin signaling but have yet to initiate proliferation and growth.

Previous work has found that insulin signaling from glia is required for neuroblasts to exit quiescence [28]. To investigate this connection, we explored glial gene expression related to neuroblast quiescent signaling pathways. We sub-clustered from the whole atlas 11,004 cells from clusters that were positive for the pan glial marker *repo* (Fig. 2e; Supp. Table 3) [47]. We found four glial subtypes: astrocytes, cortex/chiasm, perineurial, and subperineurial glia (Fig. 2f; Supp. Table 3). Known glial-quiescent neuroblast signaling molecules were differentially regulated in the cortex/chiasm glia and surface glia between 1h and 24h when quiescent neuroblasts are re-activated. Expression of these genes was maintained in glia along with proliferating neuroblasts at later stages of larval development (Fig. 2g; Supp. Tables 4, 5 and 6). For example, Ana, a glial secreted glycoprotein that inhibits neuroblast proliferation [75], was upregulated in cortex/chiasm glia at 1h (Fig. 2g; Supp. Table 4). Insulin-like peptides (Ilps), known to be secreted by glia and promote neuroblast exit from quiescence [28], were upregulated in cortex/chiasm glia and subperineurial glia at 24h and 48h (Fig. 2g; Supp. Tables 4 and 5). Trol, a secreted molecule acting downstream of Ana to promote neuroblast proliferation [76], was upregulated in perineurial glia at 24h (Fig. 2g; Supp. Table 6).

We validated two key regulators of quiescence, InR and Foxo, by antibody staining. Both proteins are enriched in Dpn + quiescent neuroblasts in newly hatched larvae (Fig. 2h). We conclude that known regulators of neuroblast quiescence are expressed in cortex and surface glia at times consistent with a role in regulating the timing of neuroblast exit from quiescence. We postulate testable models for this neuroblast cell state transition (Fig. 2i). Our data supports the notion that quiescent neuroblasts express some, but not all, elements of the insulin signaling pathway (Fig. 2i), thereby transcriptionally priming them for rapid exit from quiescence. Furthermore, both cortex/chiasm and surface glia express *Ilp* genes, suggesting a shared role in signaling neuroblasts to exit quiescence. Future work will be required to further validate these models and regulatory pathways within larval quiescent neuroblasts.

### Proliferating neuroblasts shows candidate novel markers and temporal transcription factors

**Fig. 3:**
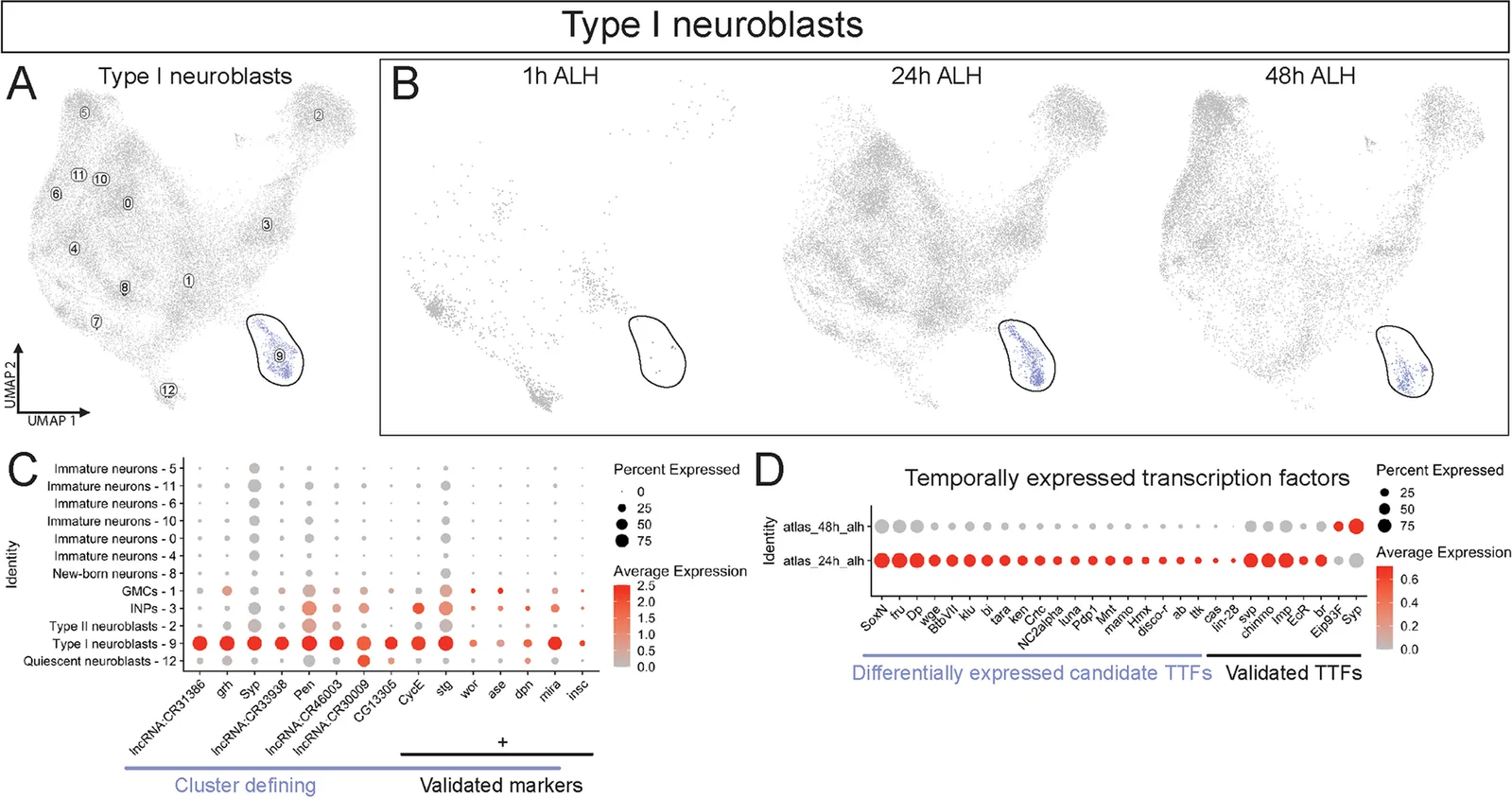
Type I neuroblasts shows candidate novel markers and temporal transcription factors. **A** UMAP of type I neuroblasts highlighted. **B** UMAP of cluster from 1h-48h ALH. **C** Dot plot of top cluster defining genes and validated markers for type I neuroblasts. **D** Dot plot of differentially expressed transcription factors between 24h ALH and 48h ALH type I neuroblasts

Here we focus on exploring gene expression in the proliferating type I and type II neuroblasts, beginning with the type I neuroblast population. We identified a type I neuroblast cluster (cluster 9; Fig. 3a,b) based on multiple validated progenitor and Type I neuroblast specific markers including: *CycE, str, wor, ase, dpn, mira* and *insc* plus lack of the type II specific marker *pointed* (Fig. 3c; Table 1; Supp. Table 2). The type I neuroblast cluster was most prominent at 24h and 48h (Fig. 3b), most likely due to neuroblasts at 1h being partitioned into the quiescence neuroblast cluster (see above). Not surprisingly, all markers except for *insc* were found to be cluster defining genes, demonstrating the robustness of the progenitor atlas in clustering by known cell type markers (Fig. 3c, right). The top cluster defining genes include known progenitor genes such as *Pen* (also called *oho31*), *grh* and *Syp* [39, 77–79]. In addition, we noticed novel genes in Type I neuroblasts that are uncharacterized such as several long non-coding RNAs and *CG13305* (Fig. 3c, left). These cluster defining genes are novel candidate markers for Type I neuroblasts.

Neuroblasts are known to have temporal transcription factor (TTF) cascades [80]. To identify novel candidate TTFs, we identified differentially expressed transcription factors between 24h and 48h type I neuroblasts (Fig. 3d; Supp. Table 7). Surprisingly, we only found candidate transcription factors upregulated at 24h (24h > 48h), but not the opposite (48h > 24h). Validated TTFs for type II neuroblasts [81, 82] show their expected trend between 24h and 48h, with the exceptions of unexpected early expression of *EcR* and *Br* at 24h compared to their published expression only after 60h [81, 82]. This could be due to the presence of mRNA but not protein (i.e. post-transcriptional regulation) or due to detection of multiple isoforms with some isoforms only expressed after 60h. Our findings identify novel candidate type I neuroblast TTFs.

**Fig. 4:**
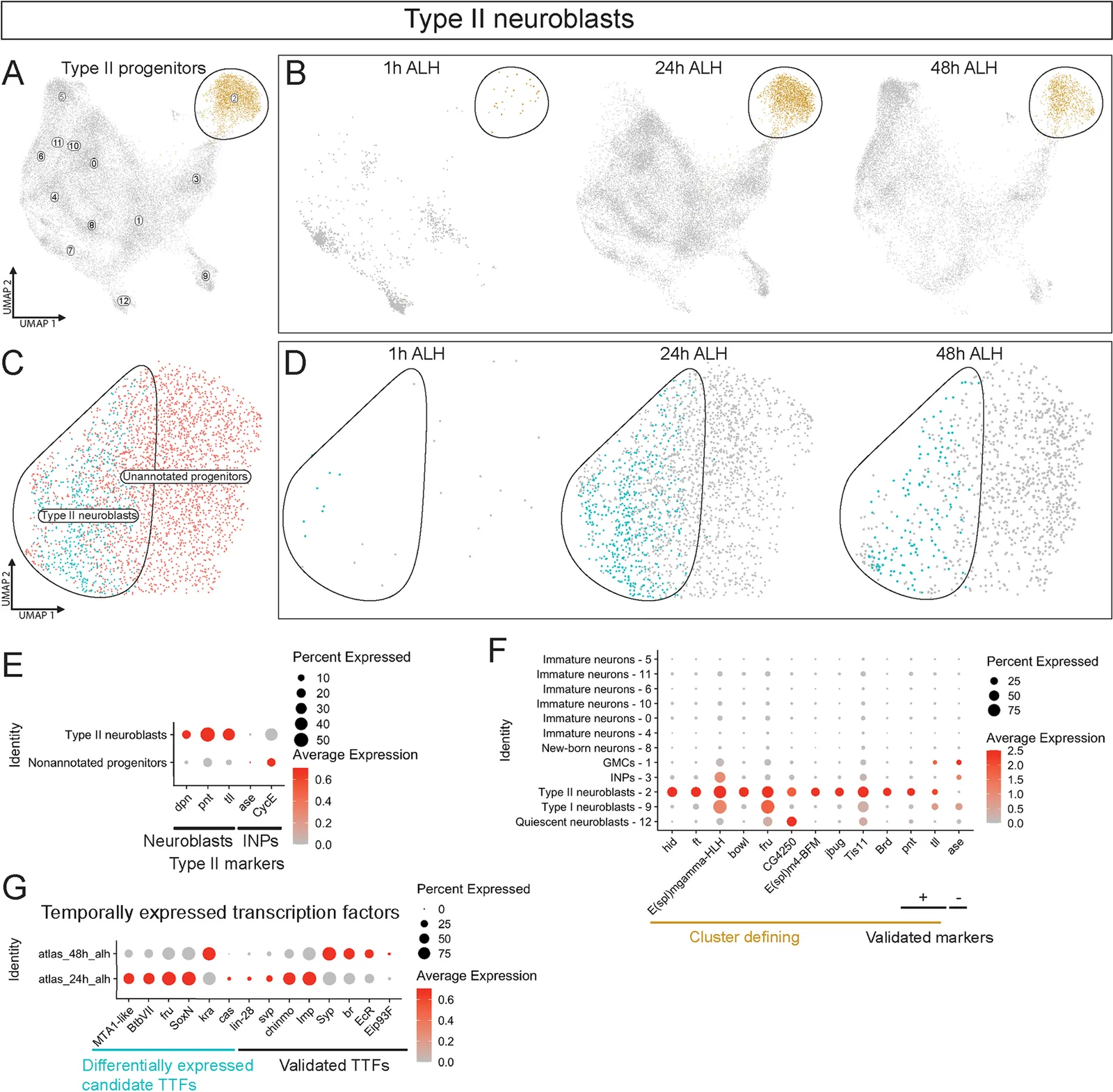
Type II progenitor cluster contains type II neuroblasts that show candidate temporal transcription factors. **A** UMAP of type II progenitors highlighted. **B** UMAP of cluster from 1h-48h. **C**  UMAP of sub clustered type II progenitors. **D** UMAP of type II neuroblasts from 1h-48h. **E** Dot plot of validated markers between type II neuroblasts and nonannotated progenitors. **F** Dot plot of top cluster defining genes and validated markers for type II neuroblasts. **G** Dot plot of differentially expressed TTFs between 24h alh and 48h type II neuroblasts

We identified a type II neuroblast cluster (cluster 2; Fig. 4a-b) based on the validated type II neuroblast markers *pnt* and *tll* with minimal expression of the negative marker *ase* (Fig. 4f; Supp. Table 2). As with the type I cluster, the type II cluster showed the most cells at 24h and 48h cells (Fig. 4b), consistent with the known type II neuroblast quiescent phase at 1h [28]. Further sub-division of cluster 2 showed two distinct clusters, one with substantially higher expression of type II neuroblast markers *pnt, tll* and *dpn* (Fig. 4e; Supp. Table 8). We identified this sub cluster as type II neuroblasts and were unable to annotate the other progenitor cluster (Fig. 4c); the unknown subcluster is not enriched for optic lobe neuroblasts nor is it enriched for low quality cells. We suspect the unannotated cluster is also composed of type II progenitors given their similarity to the type II neuroblasts and slight expression of the INP markers *CycE* and *ase* expression (Fig. 4e). These type II neuroblasts had a similar trend to type I neuroblasts in being more prevalent at 24h and 48h than at 1h (Fig. 4d). Top cluster defining genes for cluster 2 included genes specific to type II neuroblasts but also expressed in type I neuroblasts and INPs (Fig. 4f). Interestingly, the uncharacterized gene *CG4250* was exclusive to type II and quiescent neuroblasts.

We focused on identifying novel candidate TTFs between 24h and 48h in the sub-clustered type II neuroblast population. We found that validated TTFs (Fig. 4g; Supp. Table 9) followed the temporal trend previously described [80]. In addition, several novel candidate TTFs were differentially expressed between 24h and 48h (Fig. 4g). The factors *BtbVII, fru and SoxN* show expression at 24h similar to the identified Type I neuroblast candidate TTFs. Our findings identify novel candidate type II neuroblast TTFs.

### INPs express candidate novel cell type markers

**Fig. 5:**
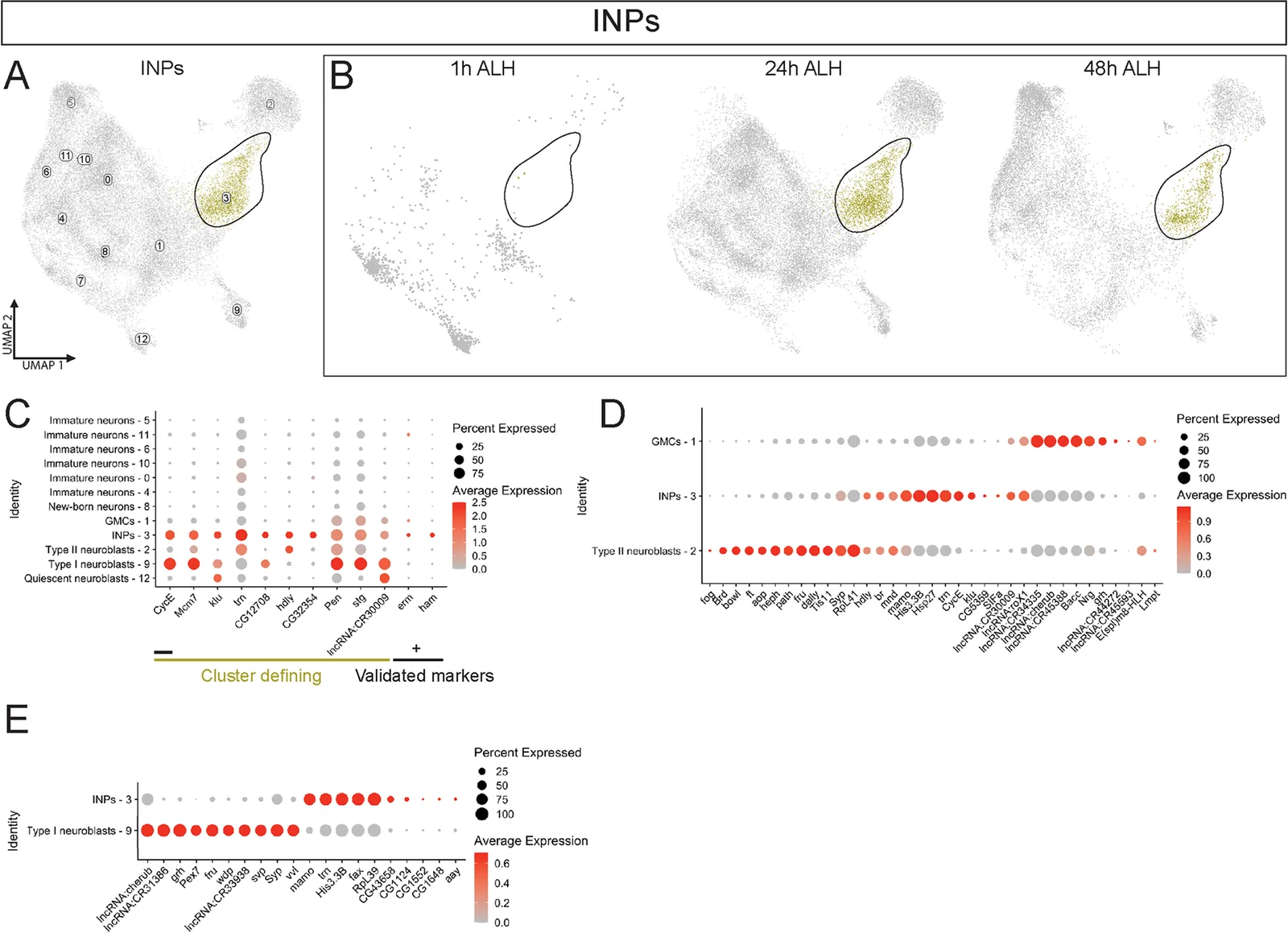
INPs show candidate novel markers. **A** UMAP of INPs highlighted. **B** UMAP of cluster from 1h-48h alh. **C** Dot plot of top cluster defining genes and validated markers for INPs. **D** Dot plot of differentially expressed genes between type II neuroblasts, INPs and GMCs. **E** Dot plot of differentially expressed genes between type I neuroblasts and INPs.

Type II neuroblasts are unique among neuroblasts by producing INPs that generate a series of 4–6 GMCs, which each produce a pair of neurons. Type I neuroblasts in the VNC and optic lobe generate GMCs, which produce just two progeny neurons. In this way, INPs are more similar to type I neuroblasts than to GMCs. INPs can be identified by the expression of general progenitor markers (*dpn*, *ase*, *mira*, *wor*) and previously validated INP-specific gene expression of *erm* (also called *fezf2*) and *ham* (Fig. 5c; Table 1; Supp. Table 2). INPs were located near the type II neuroblasts on the UMAP plots, consistent with being derived from type II neuroblasts (Fig. 5a). As expected, we detected almost no INPs at 1h (Fig. 5b, left); these are likely to be INPs produced by embryonic type II neuroblasts [83] that are in quiescence at 1h. By 24h the type II neuroblasts have exited from quiescence and have generated a pool of INPs (Fig. 5b, center) which is maintained at 48h ALH (Fig. 5b, right). We identified a number of cluster defining genes including a proliferating INP marker *CycE* (Fig. 5c). These genes are excellent candidates for selective expression in INPs and could play a role in regulating INP-specific aspects of development and function; this hypothesis awaits validating gene expression and function.

INPs are located on the “progenitor” side of the UMAP plot, nestled between their progenitor (cluster 2, type II neuroblasts) and progeny (cluster 1, GMCs; Fig. 5a). Thus, we directly compared expression of cluster defining genes for all three cell types and found clear differences in gene expression (Fig. 5d; Supp. Tables 10 and 11). We hypothesize that these genes may play a role in distinguishing the fate of all three progenitor types. INPs share a cell division pattern that is similar to type I neuroblasts (both producing a series of GMCs) as well as share expression of many pan-neuroblast genes (e.g. *dpn*, *mira, insc, wor, ase*; Fig. 1c; Table 1). Thus, we wondered how different INPs and type I neuroblasts were by scRNA-seq. We found that while many genes shared expression profiles in the two cell types, we were able to identify a number of genes that showed selective expression in INPs or type I neuroblasts (Fig. 5e; Supp. Table 12). In particular, we found several long non-coding RNAs expressed specifically in type I neuroblasts, and several previously uncharacterized genes expressed specifically in INPs. We note that *grainy head* (*grh*) is known to be expressed in both type I neuroblasts and late in some INP lineages [84–88], and it shows up as more strongly expressed in neuroblasts than INPs in our analysis (Fig. 5e). We conclude that INPs and type I neuroblasts have distinctive gene expression profiles, and that these differentially expressed genes are good candidates for distinguishing cell lineage and/or cell fate differences between these progenitors.

### GMCs, new-born neurons and immature neurons express candidate novel cell type markers

**Fig. 6:**
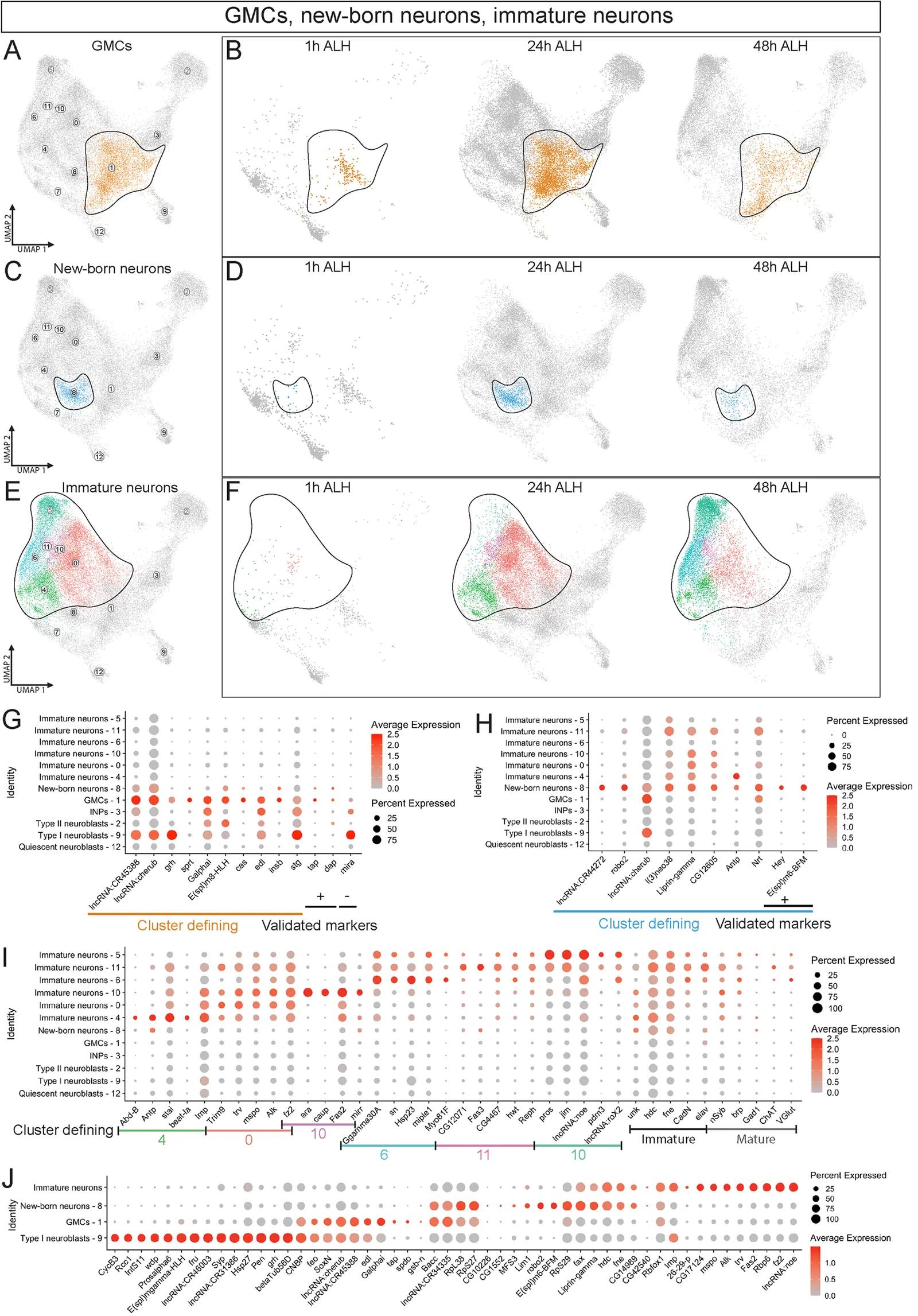
GMCs, new-born neurons and immature neurons show candidate novel markers. **A** UMAP of GMCs highlighted. **B** UMAP of GMCs from 1h-48h alh. **C** UMAP of new-born neurons highlighted. **D** UMAP of new-born neurons from 1h-48h alh. **E** UMAP of immature neurons highlighted. **F** UMAP of immature neurons from 1h-48h alh. **G**-**I** Dot plot of top cluster defining genes and validated markers for: **G** GMCs **H** New-born neurons **I** Immature neurons. **J** Differentially expressed genes between type I neuroblasts, GMCs, new-born neurons and immature neurons.

Here we focus on the more fate-restricted GMCs, derived from type I neuroblasts and INPs, and their immature neuron progeny. GMCs were positive for the validated markers *dap* and *tap;* represented in cluster 1 (Fig. 6a,g; Table 1; Supp. Table 2). We identified new-born neurons by the Notch target *Hey*, which is expressed in new-born neurons following asymmetric division of GMCs into one Notch^ON^ neuron (*Hey*+) and one Notch^OFF^ neuron (*Hey*-) [41, 89]; new-born neurons are represented in cluster 8 and include both Hey + Notch^ON^ neurons and Hey- presumptive Notch^OFF^ neurons (Fig. 6c,h; Table 1). We annotated immature neurons by the expression of published immature neuron markers and absence of mature neuron markers, and markers, and are represented in clusters 0, 4–6, 8, 10, and 11 (Fig. 6e,i; Table 1). Clusters 0 and 4 are the first immature neuron clusters to appear at 24h, closest to the new-born neurons and show the weakest expression of mature neuron markers (Fig. 6e-f,i). Conversely, clusters 5, 6 and 11 appear at 48h, are further from the new-born neurons and have the highest expression of neurotransmitters. We hypothesize that these distinct immature neuron clusters provide a differential axis given their temporal, spatial and gene marker expression patterns.

Interestingly, the three cell types (GMC, new-born neuron, and immature neuron) formed a differentiation axis from right to left in the UMAP plot (Fig. 6a,c,e); as expected, each of the three cell types were under-represented at 1h when most neuroblasts are quiescent and not producing progeny (Fig. 6b,d,f). We note that progenitors are dividing throughout larval life and add complexity to the data set given each timepoint will have each of these defined transitory cell types.

In addition to the validated cell type markers, we found potential novel markers for each cell type that drove cluster assignments. Top cluster defining genes for GMCs were shared with other progenitor cell types, but several were GMC specific including *sprt* and *cas* (Fig. 6g; Supp. Table 2). New-born neuron cluster defining genes included the validated markers *Hey* and a second putative Notch target gene *E(spl)m6-BFM* (Fig. 6h; Table 1; Supp. Table 2). The six immature neurons clusters were defined by expression of known immature neuron markers and absence of known mature neuron markers such as neurotransmitter biosynthetic genes (Fig. 6i; Table 1; Supp. Table 2).

To determine candidate novel markers that distinguish cell types along the differentiation axis, we compared each cluster for their top differentially expressed genes relative to the developmentally adjacent cell type. We grouped all 6 immature neuron clusters as a single cell type for comparison. We found distinct novel candidate markers that showed markers exclusive to individual cell types and shared between them (Fig. 6j; Supp. Tables 13, 14 and 15). Interestingly, we found type I neuroblasts and immature neurons had the most specific candidate markers (Fig. 6j, left and right) while GMCs and new-born neurons contained candidate markers shared more widely (Fig. 6j, middle). We conclude that our progenitor atlas reveals a robust gene expression along a differential axis from progenitors to immature neurons with novel candidate markers and expression profiles present in each cell type.

### Mature neurons show temporally distinct groups of transcription factors and cell surface molecules

**Fig. 7:**
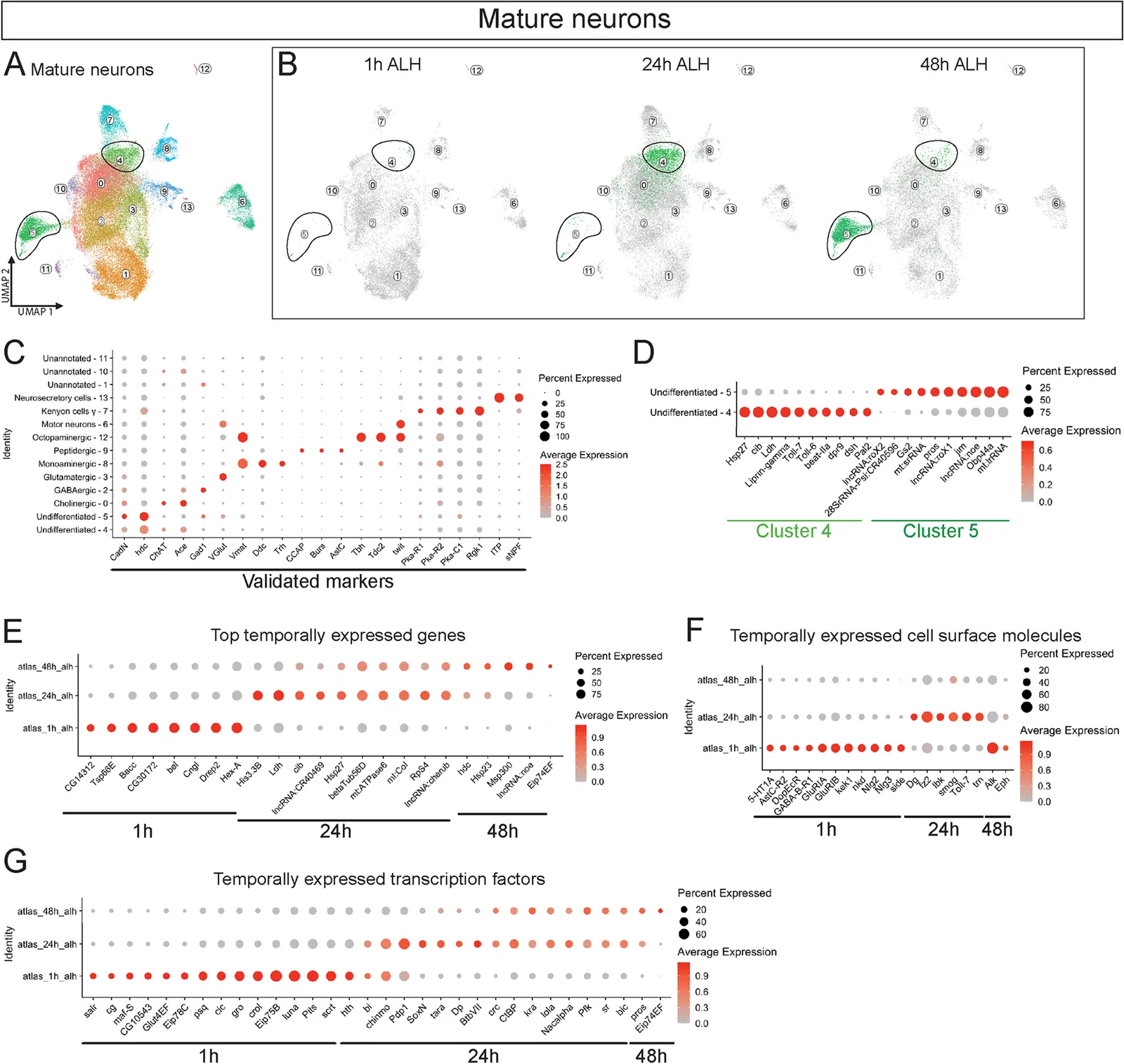
Mature neuron conclusion. **A** An atlas of mature neurons (*Brp* and *nSyb* positive) was made with a subset of 51,596 cells from the whole atlas. **B** UMAP of atlas from 1h-48h alh with clusters 4 and 5 outlined. (**C**) Validated cell identity markers label distinct neuronal cell types. **D** Dot plot of differentially expressed factors between cluster 4 and 5. **E** Dot plot of top differentially* expressed genes between 1h alh, 24h alh and 48h alh in labeled mature neurons. **F** Dot plot of differentially* expressed cell surface molecules between 1h alh and 24h alh in labeled mature neurons. 48h alh contained no differentially expressed cell surface molecule genes. **G** Dot plot of differentially* expressed transcription factors between 1h alh, 24h alh and 48h alh in labeled mature neurons. *Genes found differentially expressed in at least 2 out of 9 annotated clusters of differentiated neuron cell types.

To investigate temporal changes in mature neurons, we subclustered 51,596 cells from clusters positive for the mature neuron markers *brp* and *nSyb* from the whole atlas (Fig. 7a). Using validated markers and neurotransmitter genes, we annotated 11 out of the 14 mature neuron clusters (Fig. 7c; Supp. Table 16). Three clusters (clusters 1, 10 and 11) were left unannotated due to their lack of expression for known, validated markers (Fig. 7c, top). Surprisingly, we identified octopaminergic and neurosecretory neurons despite their relatively small cell number in the atlas of 126 and 79 respectfully. Interestingly, clusters 4 and 5 were temporally regulated, with cluster 4 enriched at 24h and cluster 5 enriched at 48h (Fig. 7b). These temporal clusters both expressed the immature neuronal markers *CadN* and *hdc*, suggesting that they are the least differentiated within this population of mature neurons (Fig. 7c, left). We further investigated the difference between the two clusters and found differential expression of cell surface molecules and neural differentiation genes such as *Toll-6/7*, *beat-IIa, jim and pros* (Fig. 7d; Supp. Table 17).

To identify temporally expressed genes within mature neurons, we focused on the remaining nine differentiated and annotated clusters within our mature neuron atlas. To circumvent the differences in cell number between clusters that may weight gene expression of larger clusters disproportionately, we found the top temporally expressed genes for each cluster and only included genes found in more than one cluster to reduce noise. We identified the top temporally expressed genes between all three time points (Fig. 7e; Supp. Tables 18, 19, 20, 21, 22, 23, 24, 25, 26 and 27). Notably, 24h and 48h neurons are more similar to each other than 1h neurons (Fig. 7e, middle), perhaps due to most 1h mature neurons being produced during embryogenesis, whereas the other clusters likely contain neurons produced during larval stages.

We further explored stage-specific differences by finding temporally expressed cell surface molecules and transcription factors in at least three out of the nine clusters (Fig. 7f,g; Supp. Tables 28 and 29). At 1h, there was an upregulation of several neurotransmitter receptors and neuroligins (Fig. 7f, left). At 24h, there was an upregulation of several cell adhesion molecules, while 48h showed no upregulation of cell surface molecule genes (Fig. 7f, middle). Interestingly, *Alk* and *Eph* were downregulated at 48h (Fig. 7f, right). We identified over a dozen transcription factors upregulated at 1h (Fig. 7g, left). Not surprisingly, 24h and 48h also were enriched for distinct groups of similar transcription factors (Fig. 7g, right). These temporally expressed genes provide novel candidates for molecules involved with dynamic roles such as synaptic wiring and neuronal function.

We found that our mature neuron atlas contains a diversity of neuronal types across all time points. We provided evidence that 1h mature neurons had more differentially expressed genes compared to 24h and 48h across top markers and transcription factors, suggesting mature neuron gene expression is more temporally dynamic prior to 24h. We conclude that we have identified candidate temporal markers within mature larval neurons.

## Discussion

Several scRNA-seq atlases of *Drosophila* larvae have been created [12, 15, 18, 20, 25, 90, 91]; however, few studies have offered multiple time points [21] but none to our knowledge have done so for the whole larval CNS as in our work (Fig. 1). Although other scRNA-seq analyses have provided and validated cell type markers [12, 15, 18, 20, 25, 90, 91], we provide novel candidate temporal factors within multiple cell types and lineages. It remains to be seen if the novel candidate markers we state here are validated *in vivo* and what their role is during development. Our work emphasizes the robustness of scRNA-seq data as supporting previously known gene expression profiles within specific cell types and providing strong candidate genes to explore. We provide access to our whole larval atlas and analysis as an easy to explore resource for the community (see Methods).

We note that some of our scRNA-seq samples had low sequencing depth and low read mapping (see Methods). Nevertheless, our whole atlas of 97,845 cells revealed a diversity of cell types: it identified all known progenitor cell types as well as many known mature neuronal types, including some that are quite rare (e.g. neurosecretory cells or insulin producing cells). The atlas contained three developmental time points (1h, 24h and 48h), and we still observed a robust differentiation axis within progenitors: from neuroblasts to neurons within UMAP plots. This further highlights the reliability of a scRNA-seq approach. In the future it would be beneficial to include additional time points across all developmental stages from embryo to adult.

### Quiescent neuroblasts and glial signaling

Neuroblasts enter a quiescent state in the late embryo and exit in the early larvae [28]. A challenge in studying quiescent neuroblasts has been the lack of cell specific markers, given their loss of canonical neuroblast markers [28, 92]. We found that quiescent neuroblasts formed a distinct cluster in the UMAP plots, the first time scRNA-seq methods have identified quiescent neuroblasts. Interestingly, the RNA-binding protein Lin-28, known to be expressed in neuroblasts at early larval stages [81, 82, 93, 94] was a cluster defining gene for quiescent neuroblasts. Lin-28 has been previously shown to play a role in regulating InR in intestinal stem cells [95]. This fits with our findings that quiescent neuroblasts are transcriptionally primed to respond to insulin signaling without expressing the cell cycle and cell growth genes that are activated upon exit from quiescence (Fig. 2h). It would be interesting to investigate other genes regulating the insulin signaling pathway as neuroblast early TTFs. It would also be interesting to test the function of the identified but uncharacterized neuroblast quiescence cluster defining genes.

Glia are known to maintain neuroblast quiescence as well as promote neuroblast reactivation via secreted signaling molecules [28, 75, 76]. As expected, we found both cortex and surface glia upregulate *ilps* at developmental times coinciding with exit from neuroblast quiescence (Fig. 2i). This provides evidence supporting the model that cortex glia express *ana* during early larval development to maintain quiescent neuroblasts while perineurial surface glia upregulate *trol* to signal an exit from quiescence. Future work should test if these glia subtypes are indeed responsible for regulating neuroblast quiescence.

### TTFs in type I and type II neuroblasts

Embryonic neuroblasts have well characterized TTF cascades [80], but it is likely that only a fraction of larval neuroblast TTFs have been identified, and even fewer have been functionally characterized. Identifying larval TTFs is complicated by larvae containing both type I and type II neuroblasts that may have similar but not identical TTFs expressed synchronously in both neuroblast populations [81, 82]. Moreover, different TTFs may be used in each type of neuroblast due to their different cell lineage (type I neuroblasts bud off GMCs while type II neuroblasts bud off INPs). Our analysis of type I and type II neuroblasts identified novel candidate TTFs with some shared and other exclusive to one of these neuroblast types. We note that identifying temporally expressed genes is difficult with only two time points, but our work should narrow the time window for validating these candidate TTFs as early expressed factors. Future work should not only explore validating these TTFs but also probing scRNA-seq data to find additional TTFs at later time points in larval development.

### Intermediate neural progenitors

INPs are produced from type II neuroblasts and add an additional TTF cascade in their divisions prior to producing GMCs [84]. Unfortunately, we were unable to provide candidate TTFs for INPs given the challenge of distinguishing INP specific TTFs from ones carried over from the type II neuroblast TTF cascade. Our analysis indicates transcriptional similarity between INPs and Type I neuroblasts with sharing common cluster defining genes (Fig. 5c). Despite the similarity between the cell types, we found differentially expressed genes that offer promising candidate genes that could underlie the different roles of these neural progenitors. This brings up an unexplored question of whether INPs and type I neuroblasts follow the same larval TTF cascade given their similarity in lineage (both produce a series of GMCs). We hypothesize that common TTFs are likely but also expect transcriptional differences that could be tested for cell type specific functions.

### The transition from progenitor to post-mitotic neurons

The transition from GMCs to newly born neurons marks a distinct developmental shift as a progenitor cell type becomes committed to a post-mitotic state. We noticed that cluster defining genes for GMCs were broadly expressed in progenitors while defining genes for new-born neurons were broadly expressed in immature neurons (Fig. 5g-h). This indicates a distinct transcriptional change captured in our analysis. We note that the GMC cluster was unexpectedly defined by *cas* expression, previously known for its expression and function in neuroblasts [96–98]; our results suggest cas should be re-evaluated for a functional role in GMCs. Future scRNA-seq work should keep in mind that candidate genes found represent transcripts not proteins; it is likely that these are not the same patterns for many genes due to post-transcriptional regulation.

Immature neurons represent an ambiguous cell identity that is poorly described in the literature, and there are few reliable markers [99, 100]. Our analysis found candidate markers that may bridge this gap. Curiously, our immature neurons were composed of six clusters; yet we were able to define it as a single cell type with the limited validated cell makers. Our cluster defining genes closely resemble those found in Michiki et al. [25] as novel neuronal markers differentially expressed over pseudotime. Additionally, our immature neuron clusters followed a developmental projection away from progenitors in both UMAP space and temporally (Fig. 1d-e). Thus, each of the six immature neuron clusters may represent discrete differentiation states within immature neurons. Alternatively, each cluster may represent neuroblast lineage-specific, segment-specific, or region-specific (e.g. central brain vs VNC). We did not observe differential expression of Hox genes in each cluster (data not shown), ruling out anterior/posterior regional clusters. Investigating how immature neurons form six discrete clusters is an interesting question for the future.

### Mature neurons show novel temporal changes

Mature neurons have been extensively studied to understand their unique neurotransmitter expression down to rare subtypes [101–105], yet limited efforts have explored temporal changes within the same neuronal identities across development. We found significant changes in gene expression across early larval development within mature neurons. Most notably, we found one neuron cluster specifically only present at 24h and a different neuron cluster only expressed at 48h (Fig. 7b). We suspect that these clusters represent larval neurons born at different times and thus become differentiated at different times. If our suggestion is correct, it would show that larval born neurons can differentiate asynchronously, rather than differentiation being triggered for all larval born neurons at a single timepoint.

Previous larval scRNA-seq datasets have characterized temporally expressed neurons within specific cell types [20, 21]. In contrast, our analysis found global temporal changes shared across almost all differentiated neurons and provided interesting candidate genes for future functional assays (Fig. 7e-g). We noticed the most significant changes occurred between 1h and 24h. Surprisingly, we found many genes encoding “mature” neuron functions were upregulated at 1h. For example, various neurotransmitter receptors and the synaptic connectivity molecules Nlg2 and Nlg3. This is likely due to the presence of embryonic-born differentiated neurons at 1h after larval hatching. These findings suggest that establishing neuronal connectivity is persisting from late embryos into newly hatched larvae.

## Conclusions

While much of the *Drosophila* genome has been extensively studied, there remains many uncharacterized genes. Our scRNA-seq analysis, similar to others [14, 24, 25, 81], can provide testable hypotheses for gene function based on cell type specific gene expression or co-expression with genes of a known function. We found many computational genes (CGs) with cell type-specific expression, as well as long noncoding RNAs. Both classes are likely to provide new insights into CNS development and function.

## Materials and methods

### Single cell isolation and sequencing

We analyzed a single-cell RNA-sequencing reads from dissociated cells collected from dissected *Drosophila* larval CNS tissue from 1h, 24h and 48h after larval hatching [27]. The raw sequencing data was obtained from GEO under the accession code GEO : GSE135810. In this study, we only used the following samples for analysis to enrich for larval neural progenitors: GSM4030593, GSM4030594, GSM4030597, GSM4030595, GSM4030596, GSM4030600, GSM4030601, GSM4030606, GSM4030602, GSM4030603, GSM4030604, GSM4030605, GSM4030607, GSM4030613, GSM4030614.

### scRNA-seq analysis

Our bioinformatic analysis was performed using Cell Ranger software (Version 6.0.1, 10x Genomics, Pleasanton, CA, USA) and the Seurat R package version 4.0.4 [106]. Briefly, Cell Ranger was used to perform demultiplexing, alignment, filtering, and counting of barcodes and UMIs, with the output being a cell-by-genes matrix of counts. Additionally, Cell Ranger was used to aggregate cells from multiple samples for each time point into single feature-barcode matrices. To further ensure that only high-quality cells were retained, we removed any cells with fewer than 200 unique features and more than 20% mitochondrial RNA.

Principal component analysis was performed with cells as samples and gene expression levels as features. The top principal components (PCs) were retained as features for downstream analyses as determined by Elbow plots. We used 50 PCs for the main atlas and most of the following clusters as this provided a compromise of significant PCs and computational cost to run downstream analyses. Based on these top PCs, cells were clustered using the original Louvain algorithm approach in Seurat. Cluster resolution was determined by optimizing clusters to fit validated markers to ensure capturing an appropriate number of cell types. In order to visualize the results of the analysis, the top PCs were used to perform a nonlinear embedding into two dimensions using the UMAP algorithm.

Differentially expressed genes within clusters were determined to be expressed in at least 10% more cells within the cluster(s) of interest compared to other clusters. Additionally, the average log fold change of expression cut off was 0.1 or more. We kept differentially expressed genes only if the adjusted p-value in a Wilcoxon Rank Sum test was below a threshold of 0.05. Dot plots show the average expression level of genes across all cells within the class. Temporally expressed genes were determined between time points in the atlas with similar number of cells. 1h cells were excluded from progenitor temporal analyses but kept with the mature neuron atlas given their approximately equal representation within the data sets.

### Subclustering for further Seurat analysis

A total of 33,458 cells were identified as either neural progenitors or immature neurons within the whole atlas based on their cluster defining gene expression of validated markers specified in Table 1. We reclustered these cells and kept 50 PCs as we did with the whole atlas and adjusted the cluster resolution to 0.49 as it provided biologically supported cell types as we identified all known progenitors with the fewest number of clusters. Differentially expressed genes were determined as described above. A total of 11,004 cells in *repo* positive clusters were labeled as glia and reclustered. We kept 50 PCs and adjusted the resolution to 0.045 as it provided the minimum number of clusters that strongly fitted known glia subtypes based on validated cell markers. We subclustered the progenitor atlas cluster 2, which we labeled as type II neuroblasts given *pnt* and *tll* expression. We kept 50 PCs and a resolution of 0.1 to show two clusters that were separated based on known cell type makers, e.g. a strong type II neuroblast cells and type II like progenitors were distinct. We subclustered 51,596 cells from *Brp* and *nSyb* positive clusters. Again, we kept 50 PCs but changed the cluster resolution to 0.37 as it provided the minimum number of clusters while capturing all known neuronal cell types that we could identify in the data.

### Data and code availability

All code used for analyses with the corresponding Cell Ranger outputs and Seurat objects are available at (https://www.dropbox.com/sh/iilbqlqysgyocbu/AADar0UdyA1Ep5qsHtRhiq_da?dl=0). scRNA-seq data is accessible under the accession code GEO: GSE135810.

### Protein localization

Standard methods were used for immunofluorescent staining [107]. The line for the foxo:GFP fusion protein is *MI00493-GFSTF.0* (BDSC#59,766) detected with anti-GFP immunofluoresence. Primary antibodies and sources: chicken anti-GFP (1:500; Abcam 13,970, Cambridge, MA, USA), rat anti-Dpn (1:100; Abcam), guinea pig anti-InR (1:500; Siegrist lab). Secondary antibodies were from Jackson ImmunoResearech and used according to product recommendation. Images were collected on a Leica SP8 laser scanning confocal microscope (Leica, Wetzlar, Germany) equipped with a 63×, 1.4 NA oil-immersion objective.

## Supplementary Information


**Additional file 1.**



**Additional file 2.**



**Additional file 3.**



**Additional file 4.**



**Additional file 5.**



**Additional file 6.**



**Additional file 7.**



**Additional file 8.**



**Additional file 9.**



**Additional file 10.**



**Additional file 11.**



**Additional file 12.**



**Additional file 13.**



**Additional file 14.**



**Additional file 15.**



**Additional file 16.**



**Additional file 17.**



**Additional file 18.**



**Additional file 19.**



**Additional file 20.**



**Additional file 21.**



**Additional file 22.**



**Additional file 23.**



**Additional file 24.**



**Additional file 25.**



**Additional file 26.**



**Additional file 27.**



**Additional file 28.**



**Additional file 29.**


## Data Availability

All data and materials will be placed in a public repository (e.g. github or Bloomington stock center) upon acceptance for publication.
